# A case of synchronous double primary combined hepatocellular-cholangiocarcinoma complicated with gallbladder adenocarcinoma: a rare case report

**DOI:** 10.3389/fonc.2026.1802177

**Published:** 2026-05-04

**Authors:** Xiangfei Wang, Linru Yang, Haiming Jian, Xiaoru Gao, Tianxiang Liu

**Affiliations:** 1 First Clinical Medical College, Gansu University of Chinese Medicine, Lanzhou, China; 2 Department of Surgical Oncology, Gansu Provincial People’s Hospital, Lanzhou, China; 3 First Department of Radiation Oncology, Gansu Provincial People’s Hospital, Lanzhou, China

**Keywords:** case report, combined hepatocellular-cholangiocarcinoma, gallbladder adenocarcinoma, multiple primary cancers, synchronous double primary tumors

## Abstract

Multiple Primary Cancers (MPCs), especially synchronous combined hepatocellular-cholangiocarcinoma (cHCC-CCA) complicated with gallbladder adenocarcinoma, are extremely rare clinically. This case report discusses a 73-year-old female patient presenting with distending pain in the right upper abdomen, nausea, and weight loss. Contrast-enhanced abdominal computed tomography (CT) and liver-specific contrast-enhanced magnetic resonance imaging (MRI) revealed a space-occupying lesion in segment S4 of the liver (considered cholangiocarcinoma) and a nodular lesion in the gallbladder wall (considered neoplastic lesion). The patient underwent open surgery, and postoperative pathology and immunohistochemical examinations confirmed: the left hemihepatic lesion was cHCC-CCA (90% moderately differentiated cholangiocarcinoma and 10% moderately differentiated hepatocellular carcinoma), the gallbladder lesion was moderately differentiated adenocarcinoma, and no cancerous tissue involvement was found at the resection margins of the liver and gallbladder. After postoperative multidisciplinary team (MDT) discussion, the diagnosis of synchronous double primary tumors of the liver and gallbladder was confirmed. The patient recovered well after surgery, and the abdominal pain symptoms were significantly improved. This is the first reported case of synchronous double primary cHCC-CCA complicated with gallbladder adenocarcinoma at home and abroad.

## Introduction

Multiple Primary cancers (MPCs) refer to two or more mutually independent malignant tumors occurring simultaneously or within an interval of 6 months in the same patient (1). In recent years, with the improvement of the popularity of national health check-ups and the advancement of imaging and pathological diagnosis technologies, the detection rate of MPCs has gradually increased. However, clinical cases of MPCs are still rare, especially synchronous double primary tumors involving the liver and gallbladder (2, 3).

The liver is a common site for MPCs, with primary tumors mainly consisting of hepatocellular carcinoma (HCC) or intrahepatic cholangiocarcinoma (ICC) (4). Combined Hepatocellular-Cholangiocarcinoma (cHCC-CCA) has histological characteristics of both hepatocellular carcinoma and cholangiocarcinoma, accounting for 1%-5% of all primary liver cancers. Its clinical phenotype and imaging features are atypical, making it easy to be confused with a single type of liver cancer (5, 6). Gallbladder cancer is mainly of adenocarcinoma as the pathological type (7). Herein, we report a 73-year-old female patient with synchronous double primary cHCC-CCA complicated with gallbladder adenocarcinoma. We analyze the clinical data, imaging findings, pathological characteristics, and treatment plan, aiming to provide a reference for the clinical diagnosis and individualized treatment of this rare disease.

## Patient information

A 73-year-old elderly female patient was admitted to the hospital due to distending pain in the right upper abdomen for more than half a year, accompanied by nausea, hiccups, acid reflux, dry mouth, and bitter taste. The patient had a 10-year history of gallstones, a history of pancreatitis episodes, and a 30-year history of hypertension. She had no history of hepatitis, denied tobacco or alcohol use, and reported no family history of liver disease or malignancy.

Physical examination: Mild jaundice of the skin and sclera; mild tenderness in the right upper abdomen, no rebound tenderness or muscle tension, and no hepatosplenomegaly palpable below the costal margin. Laboratory examinations: Carcinoembryonic antigen (CEA) was 21.82 ng/ml (normal value: <5 ng/ml), alpha-fetoprotein (AFP) was significantly elevated >2000 ng/ml (normal value: <8.78 ng/ml), CA199 and CA125 were within the normal reference range; total bilirubin, direct bilirubin, ALT, and AST were all normal. Gastroscopy and colonoscopy showed no abnormalities.

**Figure 1:**
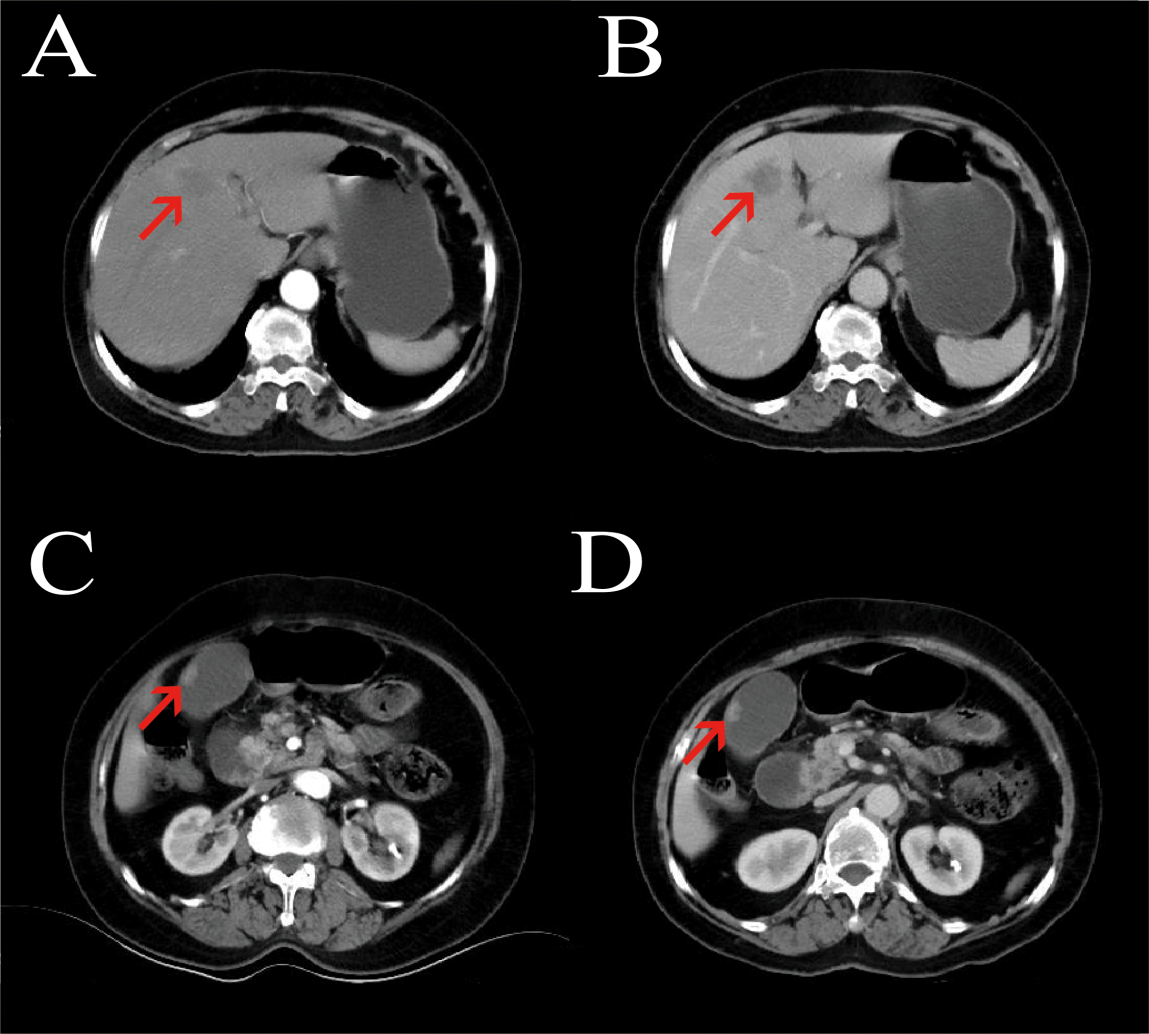
Contrast-enhanced abdominal CT. **(A)** Hepatic arterial phase; **(B)** Hepatic venous phase; **(C)** Gallbladder arterial phase; **(D)** Gallbladder venous phase.

**Figure 2:**
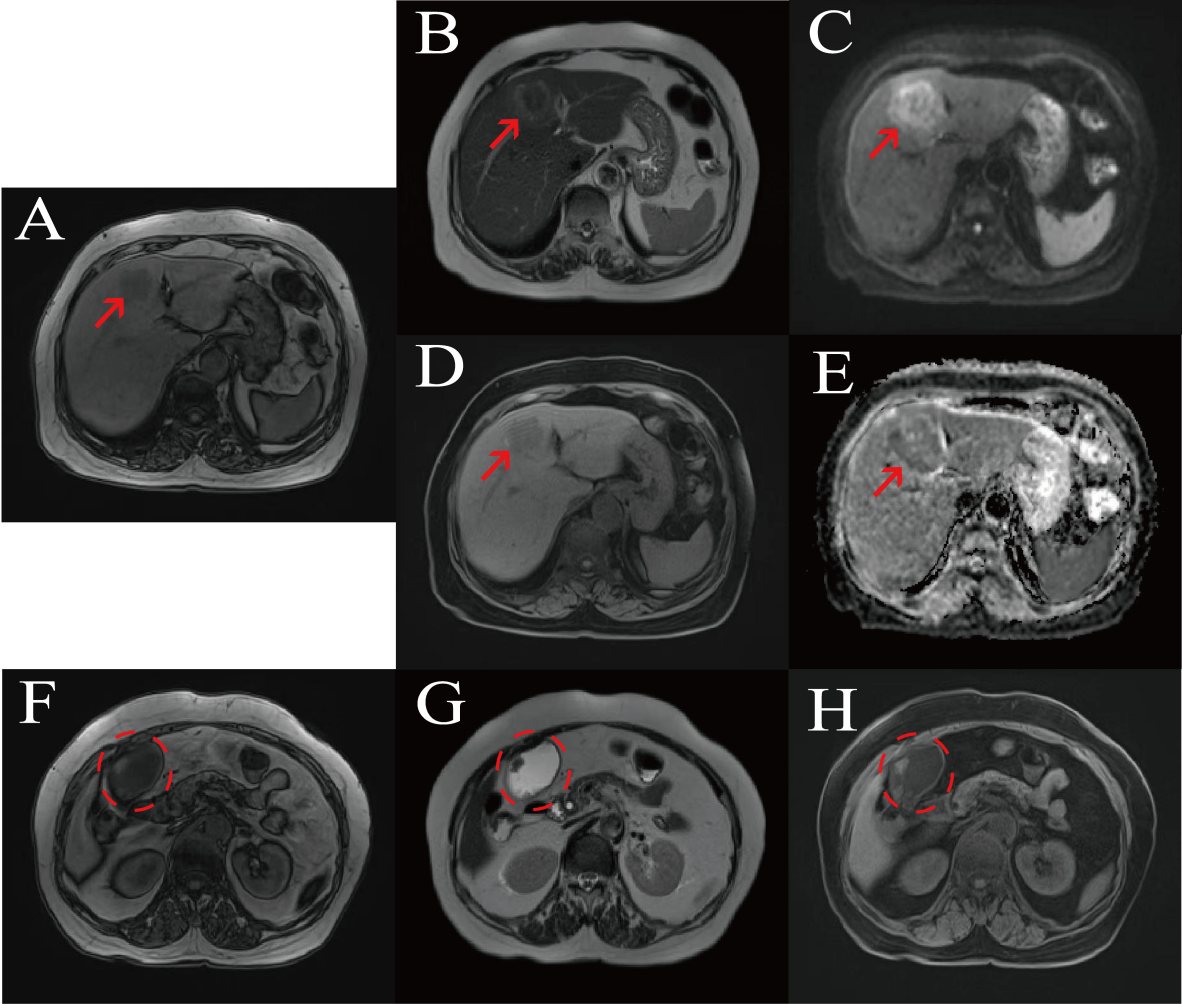
Non-contrast MRI. **(A)** T1w GRE (out-phase) sequence of the liver; **(B)** T2w sequence of the liver; **(C)** Diffusion-weighted imaging of the liver (b = 800 s/mm²); **(D)** T1w fat-saturated sequence of the liver; **(E)** ADC map of the liver; **(F)** T1w GRE (out-phase) sequence of the gallbladder; **(G)** T2w sequence of the gallbladder; **(H)** T1w fat-saturated sequence of the gallbladder.

**Figure 3:**
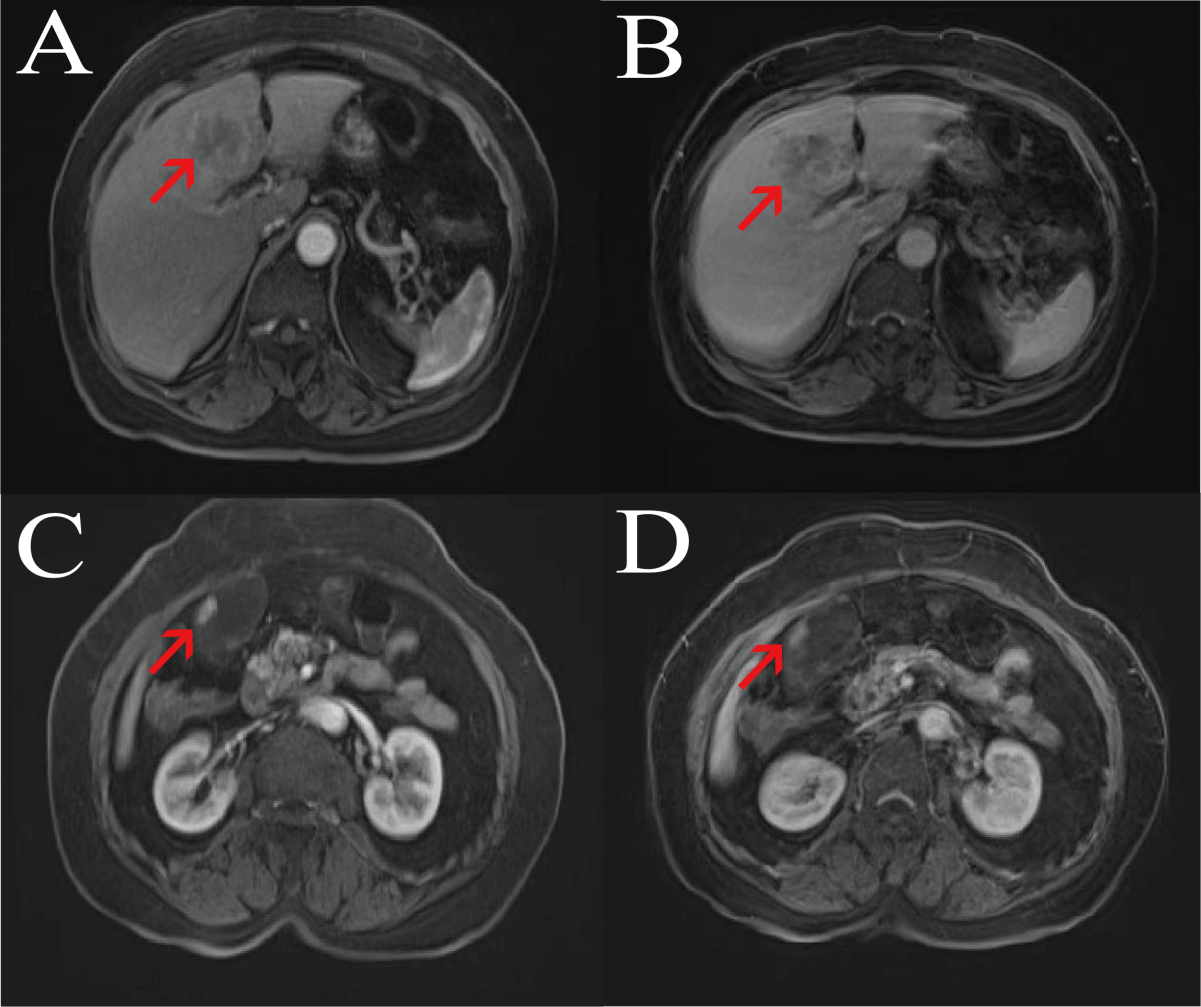
Contrast−enhanced MRI. **(A)** T1−weighted imaging of the liver in the arterial phase; **(B)** T1−weighted imaging of the liver in the portal venous phase; **(C)** T1−weighted imaging of the gallbladder in the arterial phase; **(D)** T1−weighted imaging of the gallbladder in the portal venous phase.

Imaging examinations: Contrast-enhanced abdominal CT showed an irregular mass in segment S4 of the liver (**Figure 1**), with a size of approximately 54 mm × 33 mm, ill-defined borders, and heterogeneous continuous progressive enhancement on enhanced scanning, suggesting a possible neoplastic lesion, which was considered to be cholangiocarcinoma; the gallbladder was enlarged, and a nodular lesion with obvious enhancement was seen in the gallbladder wall, protruding into the lumen, with a size of approximately 16 mm × 11 mm, considered a neoplastic lesion. Liver-specific contrast-enhanced MRI, performed using a Siemens 3.0−T MR scanner with the liver−specific contrast agent gadolinium ethoxybenzyl diethylenetriamine pentaacetic acid (Gd−EOB−DTPA), revealed an irregular mass in hepatic segment 4. The lesion showed ill−defined margins and heterogeneous signal intensity: its peripheral portion was hypointense on T1−weighted and hyperintense on T2−weighted images, while the central region was isointense on T1−weighted and hypointense on T2−weighted images, with restricted diffusion. On dynamic contrast−enhanced imaging, the mass displayed progressive, persistent, and heterogeneous enhancement, highly indicative of cholangiocarcinoma. The gallbladder was distended, and multiple nodular lesions showing hypointensity on both T1−weighted and T2−weighted images were visualized within the gallbladder lumen (**Figures 2**, **3**).

Preoperatively, liver function was assessed via routine biochemical tests and imaging examinations, combined with the Child–Pugh classification. The patient exhibited well−preserved hepatic functional reserve (Child–Pugh class A), and no radiological evidence of liver cirrhosis or fibrosis was identified. In line with the fundamental principles of hepatobiliary surgery, preservation of an intact contralateral hemiliver ensures sufficient hepatic functional reserve in patients without underlying liver disease. Given that the lesion was located in the left hemiliver, left hemihepatectomy was planned. The right hemiliver was structurally intact and unremarkable, and the estimated remnant liver volume was deemed adequate to meet the demands for postoperative hepatic functional compensation.

**Figure 4:**
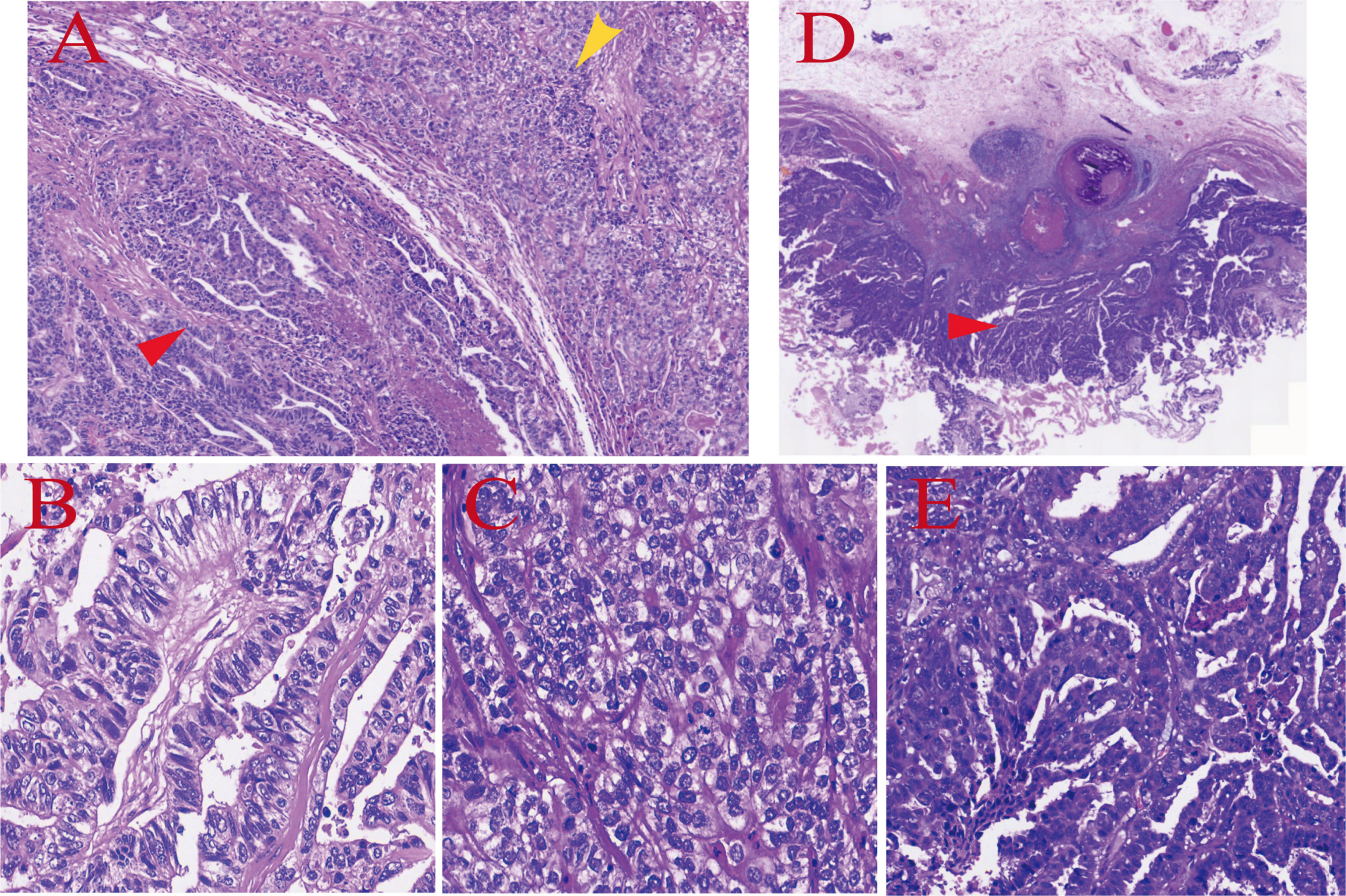
Pathological findings. **(A–C)** were obtained from the same histological section, while panels **(D, E)** were derived from another histological section. **(A)** Hematoxylin and eosin (HE) staining, ×4 magnification: Two distinct malignant tumor components with a clear boundary and no obvious infiltration or fusion were observed in the hepatic tissue, forming a mixed lesion. The red arrow indicates the cholangiocarcinoma-predominant region, and the yellow arrow denotes the hepatocellular carcinoma-predominant region. **(B)** HE staining, ×20 magnification: Tumor cells were polygonal with abundant cytoplasm and large, hyperchromatic nuclei, arranged in dense nested and trabecular patterns with sparse stroma, consistent with the histological features of moderately differentiated hepatocellular carcinoma. **(C)** HE staining, ×20 magnification: Tumor cells were cuboidal or columnar with enlarged, hyperchromatic nuclei, arranged in irregular glandular and tubular structures accompanied by abundant fibrous connective tissue stroma around the ducts, consistent with the histological features of moderately differentiated cholangiocarcinoma. **(D)** HE staining, ×4 magnification: The normal mucosa-muscularis-serosa architecture of the gallbladder wall was destroyed by adenocarcinoma tissue. Originating from the gallbladder mucosa, the tumor grew invasively into the muscularis and serosa layers in a papillary and glandular tubular pattern, with a clear boundary with the surrounding normal hepatic tissue, and no bile duct-derived lesion was detected. **(E)** HE staining, ×20 magnification: Tumor cells were tall columnar with abundant, slightly basophilic cytoplasm and basally located large, hyperchromatic nuclei, arranged in branching papillary and irregular glandular structures with secretions in some lumina. The stroma showed mild fibrous hyperplasia with mild infiltration of chronic inflammatory cells, without the characteristic desmoplastic reaction of cholangiocarcinoma, consistent with the histological features of moderately differentiated gallbladder adenocarcinoma.

The patient underwent “left hemihepatectomy + cholecystectomy + abdominal lymph node dissection” on the 10th day after admission. Intraoperative exploration confirmed that the mass was located in the left hemihepatic, the gallbladder was enlarged and adherent to surrounding tissues, and enlarged lymph nodes were seen at the porta hepatis, which were completely removed. Representative images of the surgical specimens and histopathological findings are shown in **Figure 4**. Postoperative pathological results showed: (Left hemihepatic) combined hepatocellular carcinoma and cholangiocarcinoma, specifically 90% moderately differentiated cholangiocarcinoma and 10% moderately differentiated hepatocellular carcinoma components; the cancerous tissue involved the liver capsule, two satellite nodules were visible, the surrounding liver tissue was accompanied by steatosis, and no cancerous tissue involvement was found at the liver resection margin. (Gallbladder) moderately differentiated adenocarcinoma, the cancerous tissue invaded the entire thickness of the gallbladder wall, and no cancerous tissue involvement was found at the gallbladder resection margin. Immunohistochemical results showed: CK19 (+), CAIX (focally positive in cholangiocarcinoma component), P53 (cholangiocarcinoma component: missense mutation type; hepatocellular carcinoma component: wild type), AFP (positive in hepatocellular carcinoma component), Glypican-3 (positive in hepatocellular carcinoma component), HepPar-1 (+), CD34 (sinusoidal vascularization), CEA (focally positive), Ki67 (hot spot area: 70%), MSH2 (+), MSH6 (+), MLH1 (+), PMS2 (+).

The final diagnosis was synchronous double primary combined hepatocellular-cholangiocarcinoma complicated with gallbladder adenocarcinoma. The patient recovered well after surgery, the symptoms of abdominal distension and pain were significantly improved compared with before, and the jaundice of the skin and sclera subsided. Based on MDT discussion and recommendations from the latest NCCN Guidelines, a postoperative adjuvant regimen combining chemotherapy and immunotherapy was established, consisting of sintilimab, oxaliplatin, and capecitabine. The treatment was administered in 3−week cycles: on day 1 of each cycle, sintilimab 200 mg and oxaliplatin 200 mg were given by intravenous infusion; capecitabine 1.5 g was administered orally twice daily (morning and evening) on days 1–14, followed by a 7−day rest period per cycle. A total of 4 standardized treatment cycles were completed. No obvious severe adverse events occurred during treatment, and the patient showed favorable tolerance. After the 4 cycles of treatment, follow−up examinations revealed that tumor markers including CEA and AFP decreased to within normal reference ranges. Contrast−enhanced CT of the upper abdomen and liver−specific MR imaging showed no evidence of tumor recurrence or metastasis.

## Discussion

The diagnosis of this case strictly followed the diagnostic criteria for MPCs proposed by Warren and Gates (8): (1) Both the liver tumor (cHCC-CCA) and the gallbladder tumor (moderately differentiated adenocarcinoma) were pathologically confirmed to have definite malignant characteristics; (2) The liver mass was located in segment S4 of the left hemihepatic, and the gallbladder lesion was located in the gallbladder wall; the two positions were mutually independent without continuous invasion, which conformed to the “lesion separation” principle; (3) Through imaging examinations (whole-body CT showed no other primary or metastatic tumors) and pathological morphological analysis, the possibility of metastasis or recurrence of a single tumor was excluded. Postoperative pathology of the present case confirmed that the hepatic lesion was cHCC−CCA, containing a distinct 10% HCC component that showed positive immunohistochemical staining for AFP, Glypican−3, and HepPar−1. Given that gallbladder adenocarcinoma lacks the biological potential for hepatocellular differentiation, the presence of an HCC component serves as the gold standard for excluding metastatic carcinoma and confirming primary hepatic malignancy. With respect to specific elevations in serum tumor markers: the patient presented with markedly increased preoperative AFP levels, whereas CA19−9 remained within the normal reference range. A high serum AFP concentration is highly specific for primary hepatic malignancies containing an HCC component, whereas isolated metastatic gallbladder carcinoma rarely induces such a prominent elevation of AFP. Radiologic findings demonstrated that the mass in hepatic segment 4 and the lesion involving the gallbladder wall were spatially independent, with no evidence of continuous infiltration between the two sites. The hepatic lesion exhibited heterogeneous, progressive, and persistent enhancement, consistent with the imaging characteristics of cHCC−CCA. No additional primary tumors were detected on systemic evaluation, further supporting the diagnosis of synchronous double primary malignancies.

According to the different occurrence times of each primary tumor, MPCs can be divided into synchronous and metachronous. Synchronous MPCs refer to the discovery of a second or multiple mutually independent primary malignant tumors in the same patient within 6 months after the diagnosis of the initial primary tumor; those discovered more than 6 months later are metachronous MPCs (9). Therefore, the diagnosis of synchronous double primary tumors can be clearly confirmed. Secondly, MPCs occurring simultaneously in the liver and gallbladder are extremely rare (10). In the reported literature, the histopathologically confirmed liver tumors are mostly hepatocellular carcinoma, followed by intrahepatic cholangiocarcinoma, while cHCC-CCA is rare (11), but no cases of synchronous occurrence of cHCC-CCA and gallbladder adenocarcinoma have been found. This case is the first reported case of combined hepatocellular-cholangiocarcinoma complicated with gallbladder adenocarcinoma in the literature.

Up to now, the pathogenesis of MPCs has not been fully studied. It is currently believed to be closely related to genetic factors, environmental exposure, lifestyle, and chronic inflammatory stimulation (12). In addition, liver cancer is usually associated with viral hepatitis infection, aflatoxin exposure, long-term drinking and other factors (13); while the occurrence of gallbladder cancer is related to female gender, obesity, gallstones (especially those with a diameter >3 cm or multiple stones), and chronic infections such as Salmonella or Helicobacter pylori (HP) (14, 15). The patient in this case only had a 10-year history of gallstones, and no other clear risk factors related to MPCs or hepatobiliary tumors. The patient’s 10−year history of cholelithiasis may have triggered synergistic gene mutations in gallbladder epithelial cells and intrahepatic progenitor cells through chronic mechanical irritation and sustained release of inflammatory cytokines such as interleukin−6 (IL−6) and tumor necrosis factor−α (TNF−α). Molecular characterization further confirmed the heterogeneity and independence of the lesions. The hepatic cHCC−CCA displayed a distinct dual−region pattern: the cholangiocarcinoma component harbored a missense mutation of TP53, whereas the hepatocellular carcinoma component was TP53 wild−type. This asymmetric TP53 status, together with the zonal expression of AFP and CK19, verified the nature of the mixed carcinoma at the molecular level. Although gallbladder adenocarcinoma and the hepatic lesion shared biliary epithelial markers including CK19, the unique biphenotypic differentiation and TP53 heterogeneity of the hepatic lesion were in sharp contrast to the uniform molecular phenotype and lymphatic metastatic characteristics of gallbladder adenocarcinoma, strongly excluding the possibility of hepatic metastasis. Given the high proliferative activity of the tumor (Ki−67 index: 70%) and its complex molecular background, sintilimab combined with the XELOX regimen (16) was selected in accordance with the NCCN Guidelines for Biliary Tract Cancers (17), even in the context of microsatellite stability (MSS), aiming to minimize the risk of postoperative recurrence via the synergistic effects of immunotherapy and chemotherapy.

This case is unique due to the rare combination of tumor types—synchronous double primary cHCC-CCA and gallbladder adenocarcinoma, which is the first such case reported at home and abroad. As a relatively rare subtype of liver cancer, cHCC-CCA has biological characteristics of both HCC and ICC, and its imaging manifestations often show heterogeneity due to the different proportions of the two components (18). In this case, the contrast-enhanced abdominal CT showed “heterogeneous continuous enhancement”, which was similar to the imaging characteristics of ICC, but it was difficult to distinguish from single ICC, which also reflects the challenge of preoperative imaging diagnosis of cHCC-CCA. In addition, cHCC-CCA has the characteristics of high vascular invasion and gallbladder cancer has the characteristics of early lymph node metastasis (19, 20). The superposition of the malignant biological behaviors of the two tumors may lead to a significantly faster tumor progression rate than a single hepatobiliary tumor (21).

Owing to the paucity of reported cases, there is currently no standardized treatment regimen for patients with MPCs (22). The core of treatment for synchronous MPCs is individualized precision treatment combined with MDT. For MPCs patients in the early and locally advanced stages, radical surgical resection is still the preferred option at present, and the overall survival of patients mainly depends on the tumor with the highest degree of malignancy (23). It is generally believed that after adequate and active diagnosis and treatment, the prognosis of MPCs patients is not necessarily worse than that of patients with a single primary tumor (24). The patient in this case underwent tumor surgical resection, and the postoperative resection margins were all negative, meeting the R0 resection standard. The patient recovered well after surgery, and the abdominal distension and pain were significantly improved. Considering that the patient’s gallbladder cancer had developed lymph node metastasis (pN1) and cHCC-CCA has a high degree of malignancy, the MDT recommended postoperative genetic testing to evaluate the suitability of chemotherapy combined with targeted therapy or immunotherapy, so as to reduce the risk of recurrence and improve the long-term prognosis.

This case has certain limitations: (1) Single case, lack of large-sample data support, and the universality of the conclusion is limited; (2) The postoperative follow-up time of the patient is only 4 months, so long-term survival and prognosis-related outcomes such as tumor recurrence and metastasis cannot be observed.

## Conclusion

This case is the first reported case of synchronous double primary cHCC-CCA complicated with gallbladder adenocarcinoma at home and abroad, which belongs to a rare type of MPCs. Preoperative diagnosis is very important for MPCs, including imaging and tumor markers. However, due to the rarity and particularity of cHCC-CCA, the limitation of preoperative imaging diagnosis is highlighted. The definite diagnosis of the disease still relies on pathological and immunohistochemical results. For such rare diseases, the treatment strategy should focus on the tumor with the highest malignancy, and comprehensive consideration should be given to the patient’s overall condition and MDT collaboration. Radical surgical resection is the main recommended treatment for MPCs patients. After surgery, adjuvant therapy such as chemotherapy, targeted therapy, or immunotherapy can be given according to the pathological characteristics of the tumor and genetic testing results to further improve the curative effect.

In addition, clinicians should be alert: For patients with chronic hepatobiliary diseases (such as gallstones), long-term follow-up, regular screening of tumor markers and imaging examinations are recommended. For hepatobiliary space-occupying lesions with atypical imaging manifestations, pathological and immunohistochemical examinations should be improved as soon as possible to avoid missed diagnosis or misdiagnosis. Individualized treatment and MDT are extremely important in the diagnosis and treatment of rare cases, which can significantly improve the prognosis of patients.

## Data Availability

The original contributions presented in the study are included in the article/supplementary material. Further inquiries can be directed to the corresponding author.
